# Feasibility of a birth-cohort in Pakistan: evidence for better lives study

**DOI:** 10.1186/s40814-022-00980-x

**Published:** 2022-02-07

**Authors:** Yasmeen Anwer, Fahad Abbasi, Ariba Dar, Abdullah Hafeez, Sara Valdebenito, Manuel Eisner, Siham Sikander, Assad Hafeez

**Affiliations:** 1 Present Address: Global Health Department, Health Services Academy, Islamabad, Pakistan; 2 Instititute of Criminology, University of Cambridge, Cambridge, UK; 3 Global Institute of Human Development, Shifa Tameer-e-Millat University, Islamabad, Pakistan

**Keywords:** Feasibility, Birth Cohort Study, Pakistan, Maternal and child wellbeing, Maternal Mental Health Evidence for Better Lives Study (EBLS), Foundational research, EBLS-FR

## Abstract

**Background:**

Evidence for Better Lives Study Foundational Research (EBLS-FR) is a preliminary endeavor to establish the feasibility of a global birth cohort, and within this feasibility study, piloting the research instrument, with participants from eight lower middle-income countries across the globe. It aims to investigate mediators and moderators of child development and wellbeing; it envisages informing policy and practice change to promote child health and wellbeing globally. Pakistan is one of the resource poor lower middle-income country (LMIC) taking part in this global birth cohort; we report the feasibility of establishing such a birth cohort in Pakistan.

**Method:**

From March 2019 to July 2019, 153 third trimester pregnant women were identified, using community health worker registers, and approached for baseline demographics and a number of maternal wellbeing, mental health, support-related information, and stress-related biomarkers from bio-samples in a peri-urban area of Islamabad Capital Territory. One hundred fifty of these women gave consent and participated in the study. From October 2019 to December 2019, we re-contacted and were able to follow 121 of these women in the 8–24 weeks postnatal period. All interviews were done after obtaining informed consent and data were collected electronically.

**Results:**

One hundred fifty (98.0%) third trimester pregnant women consented and were successfully interviewed, 111 (74.0%) provided bio-samples and 121 (80.6%) were followed up postnatally. Their mean age and years of schooling was 27.29 (SD = 5.18) and 7.77 (SD = 4.79) respectively. A majority (82.3%) of the participants were housewives. Nearly a tenth were first time mothers. Ninety-two (61.3%) of the women reported current pregnancy to have been unplanned. Overall wellbeing and mental health were reported to be poor (WHO-5 mean scores 49.41 (SD = 32.20) and PHQ-9 mean scores 8.23 (SD = 7.0)). Thirty-eight (21.8%) of the women reported four or more adverse childhood experiences; 46 (31.3%) reported intimate partner violence during their current pregnancy. During the postnatal follow up visits, 72 (58.0%) of the women reported breastfeeding their infants.

**Conclusion:**

The foundational research demonstrated that Pakistan site could identify, approach, interview, and follow up women and children postnatally, with a high response rates for both the follow up visits and bio-samples. Therefore, a future larger-scale pregnancy birth cohort study in Pakistan is feasible.

**Supplementary Information:**

The online version contains supplementary material available at 10.1186/s40814-022-00980-x.

## Key messages

- Pre-feasibility uncertainties existed about recruitment, contacting and re-contacting participants, consent and data collection including bio-samples, and acceptability of the methods and survey questionnaire.
- Key feasibility findings showed the ability to identify, recruit, interview, and collect varied types of biological samples. Contact with participants for follow-up interviews could be established successfully. Interview questionnaire was found acceptable by the participants.
- The feasibility findings in Pakistan imply that a birth cohort can be established with all its requisites and be part of the EBLS global birth cohort initiative.

## Introduction

### Background and rationale

Intrauterine life and early childhood are phases of rapid growth and development, with high susceptibility to myriad exposures—both helpful and detrimental to future health and wellbeing of the child [1]. Birth cohort studies are important to learn about these exposures and their subsequent impact on early and late childhood development and wellbeing. Evidence for Better Lives Study (EBLS) is one of the first initiatives in the world aiming to establish a multisite birth cohort, comprising 12,000 children across the low- and middle-income countries (LMIC), who will be followed from intrauterine life to early childhood. One of the main goals of this global cohort will be to assess a variety of maternal and environmental exposures, and their longer-term effects on children’s wellbeing and resilience [2–4].

Childhood mental health is a key determinant of later mental, physical, and social health in life [5]. Yet, it is an aspect of child development and health that is paid little attention to, especially in low-resource settings. Similarly, exposure to violence during childhood has been proven to be detrimental for children’s cognitive, emotional, and social development and overall wellbeing. Not only does violence pose an immediate risk of bodily harm for children, it also predisposes them to a cascading chain of lifelong physical, mental, and social health risks, disease, and early death [6]. However, low- and low-middle-income countries face major challenges in developing effective strategies to improve child mental health and preventing and responding to violence against children (VAC). The first step in this direction would be to have robust data about the determinants, risk factors, mediators, and moderators of VAC.

EBLS will also focus on understanding how children can reach their full potential and build resilience, and how VAC can be reduced through interventions, given their contextual realities. In the process, perhaps discovering cross-cultural, and culture-specific mechanisms underlying developmental trajectories. In summary, the biological, psychological, environmental, social, and cultural influences which shape children’s lives and inform their experiences and behaviors will be the focus of this global cohort set up across diverse sociocultural settings. In order to understand the feasibility of such a complex comparative long-term longitudinal study, a research team was started in 2018, to conduct an extensive feasibility study. It aimed at gaining experience about all major components of a future study in respect to organization, translation, ethics, fieldworker training, recruitment, and analysis. The feasibility study comprised eight study sites; Kingston (Jamaica), Koforidua (Ghana), Worcester (South Africa), Cluj Napoca (Romania), Ragama (Srilanka), Hue (Veitnam), Valenzuela (Phillipines), and Tarlai Kalan (Pakistan); with similar recruitment strategies, the same instrument, etc. The challenges faced and approaches taken in the different sites varied. In the present article, we report specifically on results of the feasibility study in Pakistan and some salient results of the instrument that was piloted [3].

### *Objectives*

The objectives of the EBLS Foundational Research (EBLS-FR) in Pakistan were to test the feasibility of managing, organizing, and operationalizing the identification, recruitment, interview, biological sample collection, and follow-up of third trimester pregnant residents of Tarlai Kalan, in coordination with eight international sites, adhering to the study protocol, and following similar methods. A secondary objective was to pilot test the instruments and analyze the data thus collected to get initial estimates of maternal wellbeing and associated exposures.

## Methods

### Setting

EBLS-FR was conducted between March and December 2019 in eight medium-sized cities, consisting of 150–800,000 residents, in eight low- and middle-income countries. This was to ensure manageability of sampling, data collection, and possible policy engagement. Tarlai Kalan, Pakistan, was one of the eight cities selected. Tarlai kalan is a densely populated, semirural, semi-literate, low-to-middle income town which is part of the Islamabad Capital Territory (ICT), with a population of approximately 150,000. Over the last decade, a sizable proportion of population have become residents of Tarlai Kalan as well as the ICT area. This influx of population over the years have originated from the neighboring provinces of Punjab and the north western province of Khyber Pakhtoonkhwa causing ICT to show a large intercensal growth rate [7]. The average household has four to eight children and mixed modes of income including irregular jobs, daily-wage work, small businesses, e.g., shops and skilled work, and government and private sector employment. The neighborhoods are conservative and relatively strict gender roles are followed [8]. Most women are housewives and extended families sharing a house or living in the same neighborhood are common. Mobility is somewhat restricted and younger women walk the streets and travel in groups or accompanied by a male family member or an older woman [8, 9].

### Study protocol

A comprehensive study protocol was followed, which was developed by the research consortium comprising of all co-investigators in the EBLS project led by Valdebenito, S. The protocol was the key document for planning and coordination of research activities across sites and ensuring comparibility. It outlined the study design, processes for data collection, management, and analysis, and ethical and project management guidelines. Annexures provided protocols for translation, information package for participants, template for informed consent, a screening questionnaire, referral form, an emergency and a fieldworkers’ safety protocol, and baseline, follow-up, and biological sample questionnaires.

### Fieldworker training and data collection

Trained research staff with postgraduate qualifications in health and social sciences screened potential participants on the eligibility criteria, conducted baseline and follow-up interviews, and collected biological samples. Site coordinators from eight countries were trained together as a group. The site coordinator in Pakistan in turn trained the fieldworkers. A detailed manual was provided by the coordinating team based at Cambridge. This contained all steps of the process, i.e., recruitment, informed consent, questionnaire administration and recording the data on tablets, biological sampling, handling computer-assisted interviews, and uploading data to a central server.

The interviews were interviewer-led computer-assisted personal interviews (CAPI) for the most part. Some sensitive questions about childhood adversity and intimate partner violence, etc. had an audio module available, where the participant could listen to the questions and response options, and record their responses themselves on the tablet (computer-assisted self-interviews or CASI). Interviewees could therefore choose to listen or read the questions and responses according to their personal preference. Interview responses were uploaded to “Qualtrics” survey software.

### Co-ordination and monitoring

Weekly site-team meetings were held to discuss progress and solve problems as they emerged. Co-ordinator and site-leaders were kept abreast of scheduling and developments either in person or through emails. Coordination with other sites and Cambridge was maintained via emails and online video conferences attended by site leaders and coordinators. The survey software used provided quality control through recording duration of interview and geo-coordinates of interview locations.

### Participants and sampling

The sampling units included 34 Lady Health Workers’ (LHWs), based catchment areas (each catchment area is based on 1200-1700 population that LHWs serve) in 12 localities of Tarlai called “Mohallas” in 8 wards, as well as the antenatal clinic at Rural Health Center (RHC) Tarlai. Each LHW’s catchment comprises 200–250 households.

As per the study protocol, 150 third trimester pregnant women were recruited through convenience sampling, and baseline data were collected. Follow-up interviews of 121 out of 150 participants were successfully done at 2 to 6 months (8–24 weeks) postpartum from October to December 2019.

### Eligibility

Women who were over 18 years of age, permanent resident of Tarlai Kalan, and at least 29 weeks pregnant at the time of interview were eligible for recruitment. In addition, a working knowledge of the Urdu language was important for informed consent. For the follow-up interview, women who had a live birth, and were between 8 and 24 weeks postpartum, were approached. Those who had a stillbirth, loss of pregnancy, or neonatal death were excluded.

### Recruitment

Recruitment first began at the Rural Health Centre Tarlai Kalan, where 34 lady health workers (LHWs) identified and brought eligible pregnant women in their third trimester. Additionally, the lady health visitor (LHV), which is a trained facility based midwife, running the antenatal clinic at the health facility (RHC) also referred eligible eligible pregnant women to the study. One hundred fifty-three potentially eligible women were approached, out of whom three refused participation. Seventy out of the 150 (46.7%) interviews were conducted in the health facility. Nineteen out of 150 (12.7%) participants were referred from the antenatal clinic in the health facility.

The above strategy of recruiting study participants at the facility that were brought in by LHWs from the community settings specifically for our study and simultaneously getting a routine antenatal check-up done proved inefficient. This was because of our contextual factors: most women needed permission for their husbands and mother-in-law for going to the health facility, as well as time and support for childcare and household chores, to step out of the house and travel to the health facility for an interview.

This was mitigated by recruiting potential participants at LHWs’ “Health Houses”^1^, located within their communities, which they serve. Eighty out of 150 (53.3%) baseline interviews were conducted in the health houses, within the community settings, as the pregnant women found this option far more feasible to come to compared to to going to the health facility. This was an efficient solution, developed with the participation of research team, LHWs, and in consultation with the coordinating team at Cambridge, with higher acceptability in the community as expressed by LHWs and study participants. Follow-up of participants who had been referred from the antenatal clinic in the health facility were contacted on phone, and the rest were invited to their respective LHWs’ Health Houses within the community settings and walking distance from participants houses.

Participants who refused follow-up, or were unavailable for it, communicated this through the LHWs. LHWs live within the same communities our participants come from and hence have ready access. Reasons for inability to participate or refusal were conveyed through LHWs or expressed directly to the research team over the phone in case of those recruited through LHV at the antenatal clinic.

### Consent

Eligible women provided a written informed consent for interview and biological samples. Consent to follow-up the mothers and their babies was also obtained at the recruitment stage. Biological samples involved dry blood spots (DBS) and hair. The informed consent was obtained by the trained research staff mentioned above, who ensured all participants understood confidentiality and their right to withdraw at any time during the study.

### Measures

#### Questionnaire development and selection of instruments

The research questionnaire was developed through an iterative process involving the larger consortium members. Instruments were selected that measured maternal wellbeing and associated risk factors during pregnancy; socio-ecological components of human development, e.g., neighborhood, family, and individual characteristics; and were cross-culturally valid and suited site-specific interests. All instruments  were translated into Urdu by two independent translators. The translations were reviewed by the site lead and modifications were made where required. A final quality check of translations was performed by site coordinators before the translated questionnaire was entered into the survey software “Qualtrics” (Table 1)

**Table 1:** Summary of measures and instruments

Constructs	Measures	Source	Scoring and interpretation	Used at	
				Antenatal	Postpartum follow up
Sociodemographic profile	Household composition and household equipment	DHS (adapted) [10]	18 items Multiple choices	•	
	Main and current occupation		6 items various: multiple choice, yes/no, open		
	Social status (Subjective)	MacArthur’s Subjective social Status Scale [11]	1 item 10-point scale; computed a subscale for participants who rated themselves 3 or Less		
	Grades passed and level of education	The Hoffmeyer-Zlotnik / Warner- Matrix of Education	2 items Number of grades passed and highest level of education attained		
Prenatal information	Prenatal information: Previous pregnancies, current pregnancy planned, current pregnancy unwanted, preconception weight, height, health conditions during current pregnancy, current pregnancy single/multiple, antenatal healthcare visits, long-term medications, rubella & tetanus vaccination, supplementation, ultrasound during pregnancy, intention to breastfeed	Reproductive health and pre-natal information Adapted from: South Asian Birth Cohort (START) [12] and the Millennium Study	21 items Various: multiple choice, yes/no, open	•	
	Attitudes toward physical punishments [13]	Attitudes toward spanking Deater-deckard et al.	5 items; Likert scale 1-5 Higher scores indicate higher endorsement		
Characteristics of the father	Cohabitation status, age of the father, education, occupation, employment status, ethnicity	Adapted from: South Asian Birth Cohort (START) [12] Millennium Cohort Study (NatCen 2003)	10 items; Various: multiple choice, yes/no, open	•	
Community characteristics	Neighborhood cohesion Neighborhood Closeness Neighborhood disorder Neighborhood ties	Neighborhood and Violent Crime Scale [14]	5 items; scored 1–4 4 items; scored 1–4 9 items; scored 1–4 2 items; scored 1–5 (total items: 20)	•	
Adverse childhood experiences	Household member alcoholic/substance user, Household member mentally ill, Household member imprisoned, Parental divorce, Household dysfunction, Emotional abuse, Physical abuse, Sexual abuse, Physical neglect	WHO ACE-IQ (adapted) Questionnaire [15]	18 items^a^; Mixed: Yes/No, 4-point Likert scale 1–4	•	
Social relationships	Partner supportiveness	Parent relationship quality and children’s behaviour [16]	5 items; 5-point Likert scale: 1-5	•	
	Intimate partner violence	WHO Multi-country Study on women Health & Domestic Violence against Women (VAWI) [17]	13 items; Lifetime prevalence: Yes/No Prevalence during last 6 months: Likert scale 1-4 Categories: Physical-6 items, emotional-4 items, sexual- 3 items	•	
	Social support	The Multidimensional Scale of Perceived Social Support [18]	12 items; Subscales: Family, friends, significant other 5-point Likert scale: 1–5	•	
Psychological traits and mental health	Well-being	WHO-5 (five) well-being Index [19]	5 items; 6-Point Likert scale: 0-5 score out of 25, then multiplied by 4: 0–100. 100 signifying best imaginable wellbeing	•	•
	Depression	Patient Health Questionnaire (PHQ-9) [20]. The Suicidal Behaviours Questionnaire-Revised	9 items 4-point Likert scale Possible scores 0-27 (Cut-off ≥ 10 for presence of depression)	•	
	Suicidality	SBQ-R [21]	1 item; 5-point Likert scale: 0–4	•	
	Perceived stress	Perceived Stress Scale [22]	10 items; 4-point Likert scale 1–4	•	
	Aggression	The Brief Aggression Questionnaire	12 items; 4 subscales of 3 items each to measure trait aggression: physical aggression, verbal aggression, anger, and hostility. 5-point Likert scale 1–5	•	
	Adult ADHD symptoms	Adult ADHD Self-Report Scale: ASRS version 1.1 (adapted) [23]	3 items; 5-point Likert scale: 1–5	•	
		Project on social Development from Childhood to Adulthood	2 items 5-point Likert scale 1–5	•	
	Self-control	Brief Self-Control scale (adapted) [24]	8 items Likert scale 1-5		
Substance use	Alcohol use, Smoking, Substance use	Alcohol, Smoking and Substance Involvement Screening Test (ASSIST) – Adapted version WHO ASSIST Working Group (2002) [25]	9 items Lifetime Prevalence: Yes/No 6-month prevalence: Likert scale 1–5	•	
Prenatal attachment	Pregnancy Related beliefs	Prenatal Attachment Inventory–Revised [26]	18 items; 4-point Likert scale 1–4	•	
Mother’s birth memories	Mother’s subjective emotional experience of birth	Birth Memories & Recall Questionnaire-adapted (BirthMARQ) [27]	5 Items; 7-point Likert scale: 1–7	˗	•
New-Born’s Constructs & Measures					
New-born’s Health & Well-being	Birth weight, Length, Occipito-frontal head circumference Mode of childbirth, Full-term/premature birth, ICU admission, Illness since birth	Norwegian Mother & Child Cohort study [28]	Various: multiple choice, yes/no, open	-	•
	Time of initiation of mother-child contact, Breastfeeding,	Questionnaire for Breastfeeding Mothers [29]	4 items; Multiple choice	˗	•

^a^Whereas other sites in EBLS used 19 out of 31 questions from the ACE-IQ questionnaire

As noted above, the outcomes of interest of EBLS-FR included initial estimates of pre-, and peri-natal exposures, e.g., perinatal stress, depression, social support, etc., which can affect child development in each site. With the aim of acquiring comparable data across all sites, validated and tested measures were used, which had been used in large multi-country studies.

### Maternal constructs and measures

#### Demographics

A modified DHS was used for demographic characteristics and socioeconomic status of participants. A visual analogue scale was used for perceived social status in the community. The MacArthur’s subjective social scale is administered through an image of a 10-rung ladder. The lowest rung represents the worst-off members in the community, and the highest rung depicts the best-off. Participants choose the rung they subjectively determine to be appropriate. Age, education, occupation, and ethnicity were recorded, as well as members of household and number of rooms in the house.

#### Prenatal information

Prenatal information is a broad-based construct; adapted from the South Asian Birth Cohort (START) and Millennium Cohort studies; comprising of items on parity, past obstetric experience, pregnancy-related health issues and diseases, Rubella and Tetanus vaccination status, multivitamin supplementation, whether the current pregnancy was planned and wanted, and antenatal visits to healthcare providers. Time of first antenatal visit, access to ultrasound facility, ongoing long-term medication intake, intention to breastfeed, and attitudes toward physical disciplining of children were also recorded.

#### Characteristics of the father (participant’s partner)

Since the main outcomes of EBLS involve children’s development under the influence of their environment, characteristics of the father were recorded, including his age, education, ethnicity, and employment status. Cohabitation status of the participant was also asked, i.e., whether living together, separated, divorced, etc.

#### Community characteristics

Neighborhood & Violent Crime Questionnaire was used to measure neighborhood safety, relationships, intergenerational support, and community cohesiveness on a 4-point Likert scale.

#### Adverse childhood experiences

WHO’s ACE-IQ was adapted for assessing participants’ exposure to violence and adversity before 18 years of age. Physical, emotional, and sexual violence, physical neglect, and household dysfunction were measured on a 4-point Likert scale. Death of a parent or guardian, parental mental health issues and substance use, and imprisonment of a household member were also recorded in a binary, “yes” or “no”, format.

#### Partner supportiveness

Items concerning partner’s supportiveness were adapted from Goldberg and Carlson’s 2014 work: “Parents’ relationship quality and children’s behavior in stable married and cohabiting families.” Five questions were scored on a 5-point Likert scale from “Never” to “Always.”

#### Intimate partner violence

WHO’s Violence Against Women Instrument (VAWI) from their multi country trial was adapted, and items about physical, emotional, and sexual violence in the prenatal period, as well as ever, were administered on a binary (“yes” or “no”), and a 4-point Likert scale.

#### Social support

The Multidimensional Scale of Perceived Social Support was employed to assess the level of support the participant felt she had in her life. Responses were on a 5-point Likert scale.

#### Psychological traits and mental health

Multiple aspects of maternal mental health and psychological traits of the mother were included as important influencers of child development.

#### Well-being

Maternal wellbeing was assessed on WHO’s Five Wellbeing Index on a 6-point scale from “All the time” to “at no time.”

### Depression and suicidality

PHQ-9 and Suicidal Behaviors Questionnaire–Revised (SBQ-R) were used to screen for depression and suicidality. Cut-off for PHQ-9 was ≥ 10. Symptoms of depression were recorded at both baseline and follow up.

#### Perceived stress

The Perceived Stress Scale with 10 items and 4-point Likert-style scale for respondents was administered to record stress levels of participants in the recent past.

#### Aggression

The brief aggression questionnaire for measuring trait aggression was used. Responses were on a 5-point, Likert scale from “never” to “always.”

#### Adult ADHD

Symptoms of adult ADHD were asked using the Adult ADHD Self-Report Scale (ASRS). We asked five questions on a 5-point Likert scale, ranging from “Never” to “Always.”

#### Self-control

Eight-items of the Brief Self-control scale were included to record dispositional self-control. Four of these addressed self-regulation, e.g., “I can resist temptations,” and four impulsiveness, e.g., “I do certain things that are bad for me, if they are fun.” Responses were on Likert-style scale from “not at all’ to “very much.”

#### Substance use

WHO’s Alcohol, Smoking, and Substance Involvement Screening Test (ASSIST) was used to assess substance use in the last 6 months during pregnancy. The items in the questionnaire had both binary and 5-point Likert scale choices.

#### Prenatal attachment

The Revised Prenatal Attachment Inventory records prenatal beliefs and attitudes toward the current pregnancy. A Likert scale of 4 responses to each item was used. Items included questions about the mother’s excitement and feelings about her baby.

#### Mother’s birth memories

Foley et al.’s Birth Memories and Recall Questionnaire (Birth MaRQ) was used to measure the participants feelings at the time of childbirth and in retrospect. Five items on a Likert-style scale offered selection on a scale of “almost always’ to “almost never.”

### Newborn’s health and well-being

#### Physical health

Physical health and wellbeing of the child was assessed from history and records of metrics at birth, e.g., birth weight, head circumference at birth, mode of delivery, time-lapse from birth to first contact with baby, illnesses since birth, etc. These were adapted from the Norwegian Mother and Child Cohort study (MoBa).

#### Breastfeeding

WHO/UNICEF’s Questionnaire for Breastfeeding Mothers was employed to assess whether the baby was exclusively breastfed, nonexclusively breastfed, or not breastfed at all.

#### Biological sampling

Dry blood spots for C-Reactive Protein (CRP) measurements were obtained by trained research staff at the end of the interview with consenting participants. CRP levels indicate systemic inflammation related to stress during pregnancy [30]. A short questionnaire was administered before blood sample was drawn to ascertain safety of the procedure. In the interest of fieldworkers’ safety, no DBS was taken from those who knew they had Hepatitis B or C .

Hair samples were collected as well, for accumulated cortisone and cortisol level measurements over the course of the pregnancy. Cortisone, in addition to cortisol, gives an estimate of the amount of protection from stress available to the growing fetus [31]. The participants were asked to answer a few questions about recent hair treatment and characteristics of their hair prior to sampling.

#### Data management and analysis

Appropriate measures were implemented to ensure access restriction of data, confidentiality, completeness, and quality. A unique ID was assigned to each participant. All data were electronically collected on tablets and uploaded to a data server sans personal identifiers. Additionally, personal identifiable data were collected and stored in hard copies locally under lock and key, for later contact during the postpartum follow up. Data cleaning and scale-construction was centrally done by the teams at University of Cambridge, UK. Data analysis was led centrally and also performed locally, as each site had privileged access to their own data.

#### Incentives

As per the EBLS-FR protocol, a hamper containing inexpensive daily use household products, e.g., milk and tea, etc., were given as a token of gratitude to all participants, regardless of the status of completion of interview.

## Results

### Feasibility

**Fig. 1:**
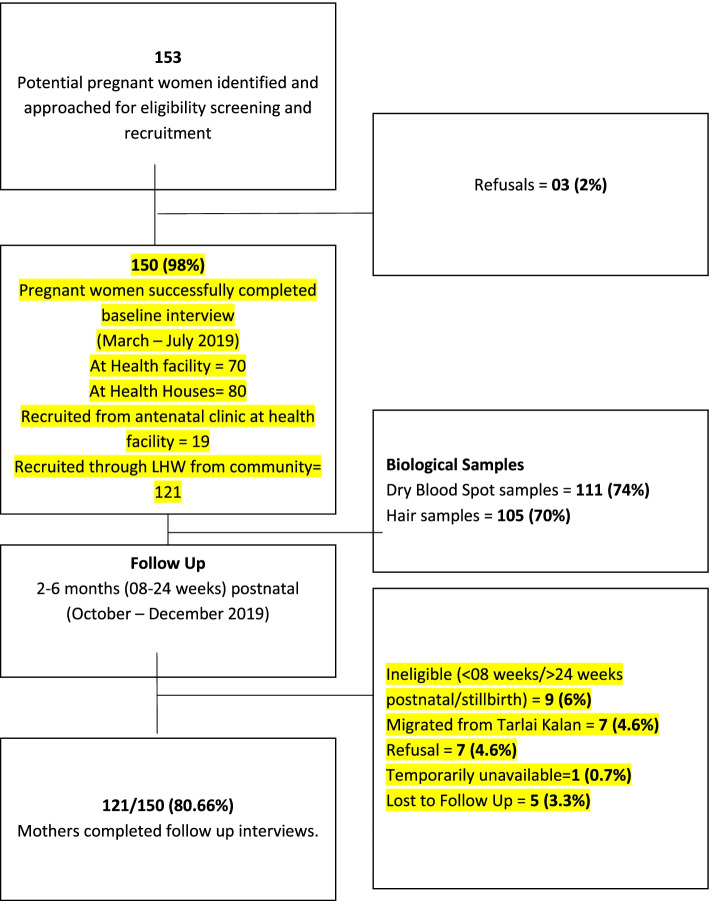
Participant recruitment and biological sampling

One hundred fifty-three potential participants were approached for recruitment by LHWs and LHV, out of which three refused to participate. One hundred thirty-one participants were recruited from the community through the LHWs registers, and 19 were referred from the antenatal clinic in the health facility. Figure 1 describes the number of participants at each stage and details of their participation. Seventy (46.6%) out of 150 baseline interviews were conducted in the health facility, 80 (53.3%) at the LHW health houses. One hundred eleven (91.7%) of the 121 follow-up interviews were conducted in health houses, nine (7.4%) in the health facility, and one (0.7%) interview was conducted at participant’s home near the health house.

One hundred eleven (74.0%) of the participants agreed to provide dry blood spot (DBS) samples, and 105 (70.0%) hair samples. Some participants refused all biological samples, others consented to one of the two types of biological samples. Commonest reasons given were fear of needle prick, time constraints, religious prohibition against clipping scalp hair for women, or unease because they had heard magic could be done with hair strands (i.e., the cultural belief of hair being used for casting magic spells and harm befalling those who would give hair strands). Novelty of taking a hair sample was also cited as a reason. Six (4.0%) participants who said they had Hepatitis B or C were excepted for DBS due to potential risks posed to the research staff handling pins.

One hundred twenty-one (80.6%) of 150 participants could be successfully followed up during the postpartum period. Out of the 29 (19.3%) of 150 who did not have a follow-up interview, eight (5.3%) were outside the eligibility range of 2 to 6 months (8–24 weeks) postnatal and one (0.7%) had a stillbirth. Seven (4.6%) had migrated from the community and could not be traced; one (0.7%) participant was temporarily away from home. Out of seven (4.6%) refusals, one (0.7%) refused due to her family’s disapproval, while the other six (4.0%) did not keep their appointment and refused an interview on further contact, citing difficulty traveling to the health facility. These six latter participants were recruited at baseline from the health facility’s antenatal clinic as opposed to the community through LHWs, and hence were more difficult to contact. Showing up for the interview required planning, time, and some costs for these participants which they mentioned on subsequent contact.

### Baseline and follow-up findings

Salient findings are summarized in Tables 2 and 3.

**Table 2:** Summary of findings at baseline *N* = 150

Baseline maternal measures (***N*** = 150)	Mean (SD) or ***N*** %
***Sociodemographic profile***	
**Age (years)**	**27.29 (5.18)**
**Education**	
Years of education	**7.77 (4.79)**
**Occupation:**	
Housewife	121 (82.3%)
Employed	26 (17.7%)
**Socio-economic status**	
McArthur Subjective Social Status Scale ≤ 3 (Range 1-10; higher score denotes perceived higher social status)	44 (29.5%)
Mean household effects (assets) Max. score: 16	6.55 (1.86)
***Prenatal health and attitudes***	
**Obstetric history**	
Parity	**2.28 (1.54)**
Nulliparous	12 (8%)
Unplanned pregnancy	**92 (62.2%)**
**Access to basic health facilities**	
Access to ultrasound	145 (97%)
At least one antenatal check-up in the 1^st^ trimester of pregnancy	120 (80%)
**Intention to breastfeed**	**148 (98.6%)**
Intended duration of breastfeeding (months)	16.50 (9.24)
**Attitudes toward physical punishment**	
Believes spanking does not harm children	37 (24.6%)
***Characteristics of the husband***	
Living with the participant (not away because of employment/divorce etc.)	138 (93.9%)
Age	32.14 (6.98)
Education	9.40 (4.80)
Husband held a paid job in last 12 months	140 (93.9%)
***Community characteristics***	
Neighborhood cohesion (1–4; higher score means more cohesion)	2.09 (0.83)
Neighborhood intergenerational closeness (1–4; higher score means more closeness)	2.22 (0.75)
Neighborhood disorder (1-4; higher score means more disordered neighborhood)	1.74 (0.62)
Drunk/intoxicated people on street	34 (23%)
Vandalism in neighborhood	30 (20%)
Litter on streets	85 (57%)
Neighborhood ties	1.93 (0.90)
Adverse childhood experiences*	
**ACE-IQ score**	**1.87 (1.90)**
Any ACE item experienced	119 (81%)
≥ 4 ACE items experienced	38 (21.8%)
Physical	39 (26.6%)
Emotional	39 (26.6%)
Sexual	7 (4.8%)
Household member violently treated	81 (55.1%)
Physical neglect	36 (24.5%)
Alcoholic/drug abuser household member	12 (8.2%)
Depressed suicidal/mentally ill household member	9 (6.3%)
Imprisoned household member	9 (6.1%)
Parents ever separated or divorced	7 (4.8%)
***Social relationships***	
**Partner supportiveness/relationship score (max score of 5—higher the score, higher the level of support)**	**4.17 (0.87)**
**Intimate partner violence frequency**	
IPV-LT (any form)	68 (46.3%)
Pregnancy-IPV (any form)	46 (31.3%)
Physical	18 (12.2%)
Sexual	13 (8.8%)
Emotional	38 (25.9%)
**Perceived social support** (mean, SD; max score of 5. Higher score indicates higher perception of support)	
Social support from family	4.06 (0.92)
Social support from friends	3.53 (1.15)
***Maternal wellbeing, mental health and other constructs***	
**Wellbeing WHO-5 (0-100, higher score means higher wellbeing)**	**49.41 (32.20)**
**Depression (PHQ-9) (mean, SD; score 0–27)**	**8.23 (7.0)**
Depressed (≥ 10)	56 (38.1%)
Moderate depression (10–14)	35 (23.8%)
Moderately severe depression (15–19)	16 (10.9%)
Severe depression (20–27)	5 (3.4%)
**Perceived stress scores (Max. score of 4. Higher scores indicate higher levels of perceived stress)**	2.14 (0.58)
Stress more than every second day	40 (27%)
**Aggression scores (1-5; higher means higher aggression)**	2.09 (0.55)
**Self-reported adult ADHD (Max. score of 5)**	1.70 (0.63)
**Self-control (max. score of 5. Higher scores indicate higher level of self-control)**	4.14 (0.58)
***Substance use***	
Lifetime prevalence	**12 (8.2%)**
6 month prevalence	**12 (8.2%)**
**Substances used**	
Tobacco	9 (6.2%)
Sleeping pills	2 (1.33%)
Other	1 (0.66%)
***Prenatal attachment***	
**Anticipation (max score of 4. Higher scores indicate higher levels of attachment)**	**2.29 (0.73)**
Almost never Imagine calling baby by name	71 (47.3%)
Almost always wonder what baby looks like	42 (28.1%)
**Differentiation (max score of 4. Higher scores indicate higher levels of attachment)**	**1.99 (0.65)**
Almost never think the baby has a personality	78 (51.7%)
Almost always thought her actions affected baby	66 (43.8%)
**Interaction (max score of 4. Higher scores indicate higher levels of attachment)**	**1.95 (0.66)**
Almost never let other people put their hands on the abdomen	128 (84.9%)
Almost always enjoy the baby move	91 (60.3%)

**Table 3:** Summary of post-natal follow up (mothers *N* = 121; babies *N* = 124)

Newborn’s health and wellbeing	
Measures	Mean (SD) or ***N*** %
Age of baby at the interview (in weeks)	**14.19 (5.09)**
< 10 weeks	29 (23.3%)
10–15	50 (40.3%)
16–20	33 (21.7%)
21–24	27 (14.5%)
Weeks pregnant at delivery	39 (2)
Preterm (37^th^ week or earlier)	15 (12.3%)
38–40	86 (71.7%)
41–42	19 (16%)
Type of delivery	
Normal vaginal delivery (Not induced)	67 (56%)
Induced and vaginally	16 (13%)
Planned cesarean	18 (15%)
Unplanned, emergency cesarean	17 (14%)
Held the baby	
Immediately	10 (8.8%)
Within five minutes	4 (3.2%)
Within half an hour	21 (17%)
Within an hour	33 (27.4%)
After C-section with general anesthesia	12 (9.6%)
Cannot remember	1 (0.8%)
After 1 h	40 (33.3%)
Gender of baby	
Boys	58 (47%)
Girls	66 (53%)
Twin pregnancy	3 (2.5%)
Birth weight (in lbs.)	6.65 (1.43)
Breastfeeding	
Breastfed exclusively	72 (58%)
Breastfed and used milk substitute	40 (32.2%)
Fed just milk substitute	10 (8%)
Mother’s wellbeing	
Mother’s birth memories-emotional (max score 35; higher score means negative emotions)	**22.67 (6.95)**
Strongly agree that:	
Had extremely positive emotions at the time of birth	15 (12.4%)
Had extremely negative emotions at the time of birth	43 (35.5%)
Had mixed positive and negative emotions at the time of birth	26 (21.5%)
Currently have extremely positive emotions when recall birth experience	24 (19.8%)
Currently have mixed positive and negative emotions when recall birth experience	33 (27.3%)
Depression PHQ-9 score	5.29 (4.27)
Depressed (≥ 10)	18 (14.9%)
Moderate depression (10–14)	13 (10.7%)
Moderately severe depression (15–19)	5 (4.2%)
Severe depression (20– 27)	0 (0%)

### Maternal constructs

As shown in Table 2, only 12 (8.0 %) of respondents with a mean age of 27.29 (SD = 5.18) were pregnant for the first time. Most already had two or more children with a mean parity of 2.28 (SD = 1.54). Participants had on an average 7.77 (SD = 4.79) years of schooling, about 2 years less than the husbands’ 9.40 (SD = 4.80) years. One hundred twenty-one (82.3%) had no personal income through employment. Most respondents had access to antenatal healthcare and ultrasound facilities. However, a majority 92 (61.3%) reported that their current pregnancy was unplanned.

Attitudes toward physical punishment of children showed slight inclination toward endorsing it, where 37 (24.6%) of the participants believed that physical punishment did not cause children any harm.

Neighborhood cohesion, intergenerational closure, and neighborhood ties were reported to be moderate. Neighborhoods were reported as relatively unsafe at a mean 1.74 score (SD = 0.62) out of 4, and unclean, with more than half of the mothers 85 (57.0%) reporting littering in the street they lived in. About a quarter, 34 (23%), said there were intoxicated people on the streets in their localities.

In all, 119 (81.0%) of respondents had experienced at least one adversity in their own childhood. Thirty-eight (21.8%) of the mothers had experienced four or more out of the listed 18 adverse experiences; over half, i.e., 81 (55.1%) had witnessed a household member being treated violently; about a quarter, 39 (26.6%) of the participants, reported physical and emotional abuse. Thirty-six (24.5%) of the participants had faced physical neglect while they were children themselves.

Most participants reported high perceived support from family, friends, and partners. However, 46 (31.3%) of the participants reported experiencing at least one form of intimate partner violence (IPV) in the last 6 months and 68 (46.3%) reported having had at least one experience in their lives. Emotional violence was most frequently reported, i.e., by 38 (25.9%), followed by physical and sexual violence by 18 (12.2%) and 13 (8.8%) participants, respectively.

Mean Patient Health Questionnaire nine items (PHQ-9) score was 8.23 (range 0–27; higher score signifies higher number and frequency of symptoms). The cut-off score for depression was 10 or higher. Fifty-six (38.1%) of the participants had depression at baseline with PHQ-9 scores above 10. However, in the postnatal period, the proportion of participants with PHQ-9 score ≥ 10 was reduced to only 18 (14.9%) of the respondents. Moreover, the severity of depression on follow-up, according to PHQ-9 scores, was found to be moderate or moderately severe (PHQ-9 scores between 10 and 19), with none of the participants scoring > 19 on PHQ-9 (severe depression). WHO’s Wellbeing Index mean score was low at 49 out of 100, signifying relatively poor perceived overall wellbeing. Only 12 (8.2%) of the participants reported substance use, with tobacco products being the most frequently imbibed substance.

Participants gave mixed responses to prenatal attachment items in all categories. Seventy-one (47.3%) of the participants never imagined calling their babies by name, and over a half, 78 (51.7%) participants did not think the baby had a personality. Yet, 66 participants (43.8%) almost always thought that their actions affected their baby. A vast majority, i.e., 128 (84.9%) participants, never allowed anyone to put their hands on their abdomen to feel the baby move; yet 91 (60.3%) almost always enjoyed feeling the baby move themselves.

On follow-up, as shown in Table 3, mean score of Birth-MARQ (emotional) was high at 22.6 (maximum 35), signifying negative emotions about childbirth experience; at 22.67 (SD = 6.95). Forty-three (35.5%) of the respondents had extremely negative emotions at the time of birth. Only 15 (12.4%) participants reported to have experienced extremely positive emotions at the time of birth. Whereas 24 (19.8%) of the mothers felt extremely positive emotions when recalling their birthing experience at the time of interview.

### New-born’s constructs

As shown in Table 3, three mothers (2.5%) had twins, increasing the total number of participating babies to 124. Fifty out of 124 (40.0%) babies were between 10 and 15 weeks of age at the time of follow-up (mean age 14.19 weeks; SD = 5.09). Eighty-six (71.7%) babies were born at term. Fifteen (12.1%) were pre- and 19 (16.1%) post-term. A total of 35 (29.0%) of the babies were delivered through a planned or an emergency cesarean section. Mean weight at birth was 6.65 lb (SD = 1.43).

Only 15 (12.0%) of the babies had been held by the mother within 5 min of birth. Furthermore, only half of all babies were held by their mothers within the first hour after birth.

In sharp contrast to the baseline of 148 (98.6%) mothers intending to breastfeed their newborns, only 72 (58.0%) could actually exclusively breastfed their babies on the follow up visit. However, partial breast feeding (breastmilk and milk substitute) was common with 40 (32.0%) of mothers supplementing milk substitute along with breastfeeding.

## Discussion

The main objective of the foundational research was to explore the feasibility of establishing a large-scale, multisite, global birth cohort in which Tarlai Kalan, Islamabad Capital Territory, in Pakistan being one of the seven other sites across the world. This involved testing the full strategy to identify, recruit, and follow up 150 pregnant women in their third trimester; pilot the instruments capturing multiple constructs, including sensitive questions; and obtain biological samples. In Tarlai Kalan, we found that we could identify, recruit, and follow up participants (with a high response rate) as well as gather biological samples from majority of our sample; our methods and strategies were acceptable to the healthcare personnel (ie LHWs and health facility staff) and participants; and that computer-assisted personal interviews did not pose any significant challenges, thus rendering this approach of data collection feasible.

However, a notable finding is that approximately quarter of our participants did not consent to provide biological samples. The most frequent reason given was the worry about getting late in returning home. This concern from women, especially from lower socioeconomic strata, with larger families to care for, and cultural norms holding them accountable for their time and activities, is not surprising in the local milieu [32]. Although less frequent, these norms exist across classes. Adeel et al. note in their analysis of Pakistan Time-Use Survey that 80% of all trips outside the house are by males in Pakistan [33]. A qualitative study in a hospital in Rawalpindi by Armaan Rowthor et al. found gender norms to be a major cause of constrained agency, decision-making, and prenatal anxiety among pregnant women [8, 32]. Other concerns about providing samples, especially hair samples, are rooted in the socio-cultural context (ie hair being used for black magic/casting evil spells etc) thus prohibiting or cautioning against sharing such biological samples [34].

We had initially planned in for LHWs to accompany participants from their communities to the health facility for recruitment into the study. This had some initial issues: sometimes, the LHWs could not accompany the pregnant women to the facility on the designated day, due to their other duties and engagements. Additionally, LHWs reported many women found it inconvenient to travel to the facility for an interview by the research team due to domestic responsibilities and concerns around permission from spouse and in-laws. Our later approach to have participants interviewed in the community-based health houses of the LHWs was welcomed by LHWs and participants for both recruitment and follow ups. This difference is not entirely unexpected considering women have limited agency and the bulk of household and caregiving responsibilities in Pakistan [8].

However, recruitment through LHWs is not without its own practical limitations. Firstly, all of the population of Tarlai Kalan is not currently covered by LHWs, leading to a potential source of selection bias. Secondly, LHWs are government employed community health workers that reside in the communities and provide preventive promotive MNCH health care and also tasked with other duties like polio vaccination, dengue, tuberculosis etc, thus scheduling interviews through the LHWs had its challenges. For example, polio vaccination campaigns which rely heavily on community workers, were a recurring activity during our study period and during these week long campaigns none of the LHWs would be available to assist the research team. We mitigated this issue by coordinating with those very few, if any LHWs who were not tasked  with polio vaccination duties. These considerations will have to be kept in view when planning a larger study in future which involves LHWs.

Contingencies for unforeseen problems must also be an in-built feature of any future prospective studies. In our study, during the postpartum follow up phase, there was an outbreak of dengue fever in Tarlai Kalan which curtailed our field activities for nearly 2 weeks. This cost us higher attrition, as some participants crossed the eligibility limit of 24 weeks postpartum.

In general, attrition was higher in the subgroup recruited directly from the antenatal clinic where six out of 19 (31.6%) participants were lost to follow up. Most frequently cited reason was difficulty leaving home and traveling to the health facility which echoed the participants whom LHWs sought to bring to the health facility [8]. Hence, we learnt that LHWs involvement improves rates of participant retention and successful follow-up.

The overall characteristics of participating mothers, and gender disparities in education and employment, closely resembled those of the general population of Pakistan as reported in PDHS 2017–2018 [35]. Respondents belonged to diverse socioeconomic backgrounds. Most respondents had regular access to healthcare and ultrasound facilities. Despite access, high unmet need for family planning among our currently pregnant sample was clearly noted, with over half of the mothers reporting unplanned pregnancies. This finding to is in-keeping with the recent Demographic and Health Survey of Pakistan [35]. The reasons, as described in surveys and studies, are varied across socioeconomic class and ethnicity. Along with lack of informed choice, power dynamics also play a role, with only about 7% of current female contraceptive users in Pakistan having made the choice themselves [8, 35].

We learned that participants reported satisfactory community and family support. Spousal support and social connectedness and support from other women in the family and community have been found to be strong enabling resources as well as important factors influencing women’s health and wellbeing [8]. Despite a high mean score of partner supportiveness IPV was experienced by 31.3% of our participants, perhaps because IPV is accepted as a normal part of a marriage in our setting [32]. IPV during pregnancy and otherwise as well as prenatal depressive symptom have been consistently found to be higher in low- and lower middle-income countries (LMICs) and especially the south Asian region [36].

High neighborhood disorder, widespread p-IPV, stress, poor wellbeing, and alarming frequency of prenatal depression are major challenges in improving maternal and child health [37]. Our findings on prevalence of perinatal depression are also comparable to earlier studies in Pakistan, reporting similar prevalence rates of depressive symptoms [38–40]. Postnatal depression was found to be markedly lower than prenatal depression, in line with local literature, which estimates the prevalence ≤ 25% [41–43]. This may point toward strong social and biological determinants of prenatal depression in the Pakistani context, although any conclusions must take into account the small sample size and 19% attrition on follow-up.

A significant proportion of the sample did not believe physical punishment harms children. This is another contextually embedded finding previously reported in literature that represents the general norms of disciplining children across our setting [44]. A history of exposure to four or more adversities in their own childhood was a frequent finding in our sample. Most frequently reported adversity was having witnessed a household member being treated violently. Scientific evidence indicates that perpetuation, victimization, and normalization of violence across generations can be an outcome of childhood exposure to violence during the early years. Hence, intergenerational transmission of violence is a real concern in societies which commonly rely on violence to address interpersonal conflict [45].

Lifetime substance use was found to be very low among our participants despite higher stress levels and depressive symptoms. This has been a consistent finding in other studies and reports from Pakistan [46, 47]. It is primarily a reflection of the cultural norms surrounding women’s acceptable behavior in semi-urban middle-class Pakistan, as well as other conservative societies. This picture is variable when high and low-income groups are studied in isolation [48]. Another factor responsible for lower substance use among women worldwide could be preferred processing mechanisms in men and women. Where men are more likely to externalize extreme stress through aggression and substance use, there is a propensity for women to internalize, leading to anxiety and mood disorders [49, 50].

Mothers’ prenatal attachment with their babies seems low at first glance, with mean values below the mid-point. Perhaps this can be explained somewhat by the fact that over half of our respondents did not plan their current pregnancy. However, another important consideration is that of the cultural context. For example, it is considered shameful for pregnant women to talk about their pregnancy and childbirth with family, or express happiness about being pregnant, since pregnancy is associated with a sexual act [51]. Beliefs about “evil eye” also deter mothers from openly expressing joy and letting people feel their baby’s movements [52]. Furthermore, in our context, women are the primary caregivers in large families. Performing caregiving work for many children and extended families living together, through the physio-psycho-social changes of pregnancy, may not be conducive for women to positively experience their pregnancy. Adding to this, other social pressures such as a strong preference for male offspring and social consequences of having many female children may lead to ambivalent feelings and stress [8]. There’s a possibility that this may be expressed as poor prenatal attachment [53].

At the postpartum follow-up, we also collected data on emotions at the time of childbirth, which were reported to be largely negative. Ahmed J. et al., in their qualitative analysis, found three main themes that could be responsible for a negative childbirth experience: The first being poor access to care during pregnancy and birth; the second, unavailability of the most appropriate care services at the time of birth; and third, and most significantly impactful in our opinion, women’s lack of agency in making timely decisions about seeking health and care. A worrying finding was that women reported being treated harshly and disrespectfully at public healthcare facilities, leading to a general avoidance of seeking medical help [54].

Only 35 (29.0%) mothers were able to hold their babies within the first half hour after birth. Guidelines on the subject ask for immediate skin-to-skin contact between mother and child, foregoing the routine practices of weighing, bathing the baby, etc. [55–57]. Almost no participant had her child’s metrics recorded at the time of birth. The only exception being the child’s birth weight—another indicator of maternal and neonatal health services in Pakistan lagging far behind international guidelines.

About one-third of the participants had childbirth through cesarean section. The trends worldwide have shown a major shift toward cesarean sections between 1990 and 2014. This trend is more marked in the high-income countries, and within all countries, in the populations falling in higher wealth quintiles [58, 59]. Pakistan is no exception, despite being a LMIC, the rising trends can be seen, with more wealth and higher education being two influential factors found to be associated with cesarean sections in Pakistan in a recent modelling study [60].

Seventy-two (58.0%) of the mothers were exclusively breastfeeding at the postpartum follow up. Forty (32.2%) reported using milk substitutes along with breastfeeding in contrast to the 148 (98.6%) who had shown intent at the baseline interview. Beliefs and myths around breast milk, e.g., mother producing “insufficient” milk, baby not being satiated with breastfeeding alone, mother’s milk being “weak”, formula milk or cow’s milk being more nutritious, etc., are widespread in Pakistan, which may have contributed to reliance on formula milk substitutes [61]. Other factors may include maternal depression, stress, resumption of household chores in the postpartum period, poor technique, lack of knowledge about the importance of breastfeeding, family physician’s advice, peers’ advice, and lack of facilities and services to initiate proper breastfeeding after birth [62, 63].

Based on our limited findings from the pilot, and based on existing scientific evidence, we tentatively propose that determinants of maternal health and child development, and maternal and child health (MNCH) services, seem to be in poor state in Pakistani low- and middle-class communities. Further research with a larger sample size is called for to better understand and meet these challenges.

In summary, the main strengths of our feasibility study were its approach to identify eligible sample using both facility-based and community-based approaches, high response rate at recruitment, postpartum follow-up and acquiring varied biological samples, using constructs informed by expert opinion through a delphi technique [64], and use of validated instruments across the eight EBLS sites making interesting cross cultural comparisons possible [3]. Specific to the Pakistan site, this study is novel in the local context in that it takes prenatal life as a period of exposure to adversity and aims to collate these findings with follow-up data over the childhood years. Our feasibility findings are promising considering the difficult context of Pakistan. Therefore, we suggest that, incorporating the lessons learned from the foundational research, a larger birth cohort can be established in Pakistan in the future [3].

## Conclusion

We believe that it is feasible to conduct a large-scale longitudinal birth cohort study which includes collecting varied types of biological samples within the Islamabad Capital Territory, Pakistan based on the lessons learnt from the current feasibility study. Recruitment during pregnancy from both facility and community settings with sensitive questions and biological samples is feasible, with greater success if done within health houses situated within community settings. Mothers, once recruited, can readily be contacted and followed up. Attrition is likely to be low with the current strategy of involving Lady Health Workers.

A prospective cohort study can help us better understand the local contextual factors impacting maternal and child health and wellbeing, and guide us towards the most appropriate steps to improve these. This will in turn help us with formulating effective strategies to improve physical, mental, and social health of mothers and children, reduce exposures detrimental to child development, and explore pathways of resilience during childhood.

## Supplementary Information

**Additional file 1: Figure 1.** Participant Recruitment & Biological Samples. Describes number of participants from one stage to the next.

## Data Availability

Further information about the datasets used and/or analyzed can be available upon request from the corresponding author.

## Footnotes

1 Lady health workers are required to designate a room in their houses for their community-based professional activities. This room stores basic supplies e.g. oral rehydrating salts, documents, and hosts children’s vaccination etc.
