# Supplementary material for: Feasibility of a birth-cohort in Pakistan: evidence for better lives study
Source: Pilot Feasibility Stud. 2022 Feb 7;8:29. doi: 10.1186/s40814-022-00980-x (PMC8819840; doi:10.1186/s40814-022-00980-x)
Supplement: Supplementary file 1 — Additional file 1: Figure 1. Participant Recruitment & Biological Samples. Describes number of participants from one stage to the next. [file 40814_2022_980_MOESM1_ESM.docx]

Figure 1 Participant Recruitment & Biological sampling

**153**

Potential pregnant women identified and approached for eligibility screening and recruitment

**121/150 (80.66%)**

Mothers completed follow up interviews.

**Follow Up**

2-6 months (08-24 weeks) postnatal

(October – December 2019)

**150 (98%)**

Pregnant women successfully completed baseline interview

(March – July 2019)

At Health facility = 70

At Health Houses= 80

Recruited from antenatal clinic at health facility = 19

Recruited through LHW from community= 121

Refusals = **03 (2%)**

**Biological Samples**

Dry Blood Spot samples = **111 (74%)**

Hair samples = **105 (70%)**

Ineligible (<08 weeks/>24 weeks postnatal/stillbirth) = **9 (6%)**

Migrated from Tarlai Kalan = **7 (4.6%)**

Refusal = **7 (4.6%)**

Temporarily unavailable=**1 (0.7%)**

Lost to Follow Up = **5 (3.3%)**
